# The effect of various pre‐treatment methods of chromium leather shavings in continuous biogas production

**DOI:** 10.1002/elsc.201900127

**Published:** 2019-11-24

**Authors:** Carolina Scaraffuni Gomes, Jens‐Uwe Repke, Michael Meyer

**Affiliations:** ^1^ Forschungsinstitut für Leder und Kunststoffbahnen (FILK) gGmbH Freiberg Germany; ^2^ Dynamik und Betrieb technischer Anlagen TU Berlin Berlin Germany

**Keywords:** anaerobic digestion, biogas production, chromium shavings, continuous trials, pre‐treatments

## Abstract

During leather manufacture, high amounts of chromium shavings, wet by‐products of the leather industry, are produced worldwide. They are stable towards temperatures of up to 110°C and enzymatic degradation, preventing anaerobic digestion in a biogas plant. Hitherto, chromium shavings are not utilized industrially to produce biogas. In order to ease enzymatic degradation, necessary to produce biogas, a previous denaturation of the native structure has to be carried out. In our projects, chromium shavings were pre‐treated thermally and mechanically by extrusion and hydrothermal methods. In previous works, we intensively studied the use of these shavings to produce biogas in batch scale and significant improvement was reached when using pre‐treated shavings. In this work, a scale‐up of the process was performed in a continuous reactor using pre‐treated and untreated chromium shavings to examine the feasibility of the considered method. Measuring different parameters along the anaerobic digestion, namely organic matter, collagen content, and volatile fatty acids content, it was possible to show that a higher methane production can be reached and a higher loading rate can be used when feeding the reactor with pre‐treated shavings instead of untreated chromium shavings, which means a more economical and efficient process in an industrial scenario.

**Abbreviations**
COD: chemical oxygen demand
DSC: differential scanning calorimetry

## 1 INTRODUCTION

The disposal of chromium leather waste is one of the most important ecological challenges in the leather industry. Many attempts to use this waste can be found in the literature but most of these wastes are still disposed off through landfill or incineration processes 1. Due to their structure composed mainly of collagen and chromium cross‐links, this solid waste is considered to be too stable for anaerobic degradation. However, Dhayalan et al. 2 studied the anaerobic digestion of chromium leather and concluded that degradation of the waste is possible using anaerobic sludge. The problem is that using this substrate leads to a very slow process and low biogas production.

Currently, there are no biogas plants in the industry using chromium leather waste as a main substrate. However, the tannery SÜDLEDER (Rehau, Germany) already has a biogas plant in operation using their own organic waste (hair, protein, fat, and chromium loaded sludge) to produce energy 3. This kind of initiative illustrates the interest of the industry in biogas production. Nevertheless, using a complex substrate as chromium leather waste needs to be further developed.

Collagen molecules are endowed with mechanical and thermal stability of the fibrous network and high stability to enzymatic degradation 4. Additionally, the chromium tanning process makes the material even more stable 5. Hence, it is necessary to denature the collagen fibres present in chromium leather waste to enable the anaerobic microorganism to degrade this solid waste 6. A pre‐treatment can be used to denature the collagen and enable degradation in order to reduce the digestion time and increase the biogas yield 7.

While some studies treated chromium leather waste under batch conditions 2, 7, 8, 9, 10, 11, 12, there is no published work on continuous reactors for this material. Continuous tests simulate long‐term process conditions and are essential for adapting the method in the industry since most large‐scale industrial digesters work in continuous mode as this allows the digester to continually produce biogas 13. These tests enable to investigate capabilities and loading limits of the process, mean residence time as well as formation and accumulation of metabolic intermediates and their influence on process stability 14.

The aim of this study is to investigate the biogas production with untreated and pre‐treated chromium leather wastes in a continuous biogas reactor. Pre‐treatment was performed by different heating and mechanical technologies.

## 2 MATERIALS AND METHODS

### 2.1 Materials

The chromium leather waste tested were chromium shavings shaved from wet chromium tanned leather. The materials were obtained from a local tannery (HEWA Leder, Freiberg, Saxony, Germany). The chromium shavings used in this work have already been air‐dried to some extent and present a water content of almost 20%.

For the biogas trials, mesophilic anaerobic sludge from the tannery SÜDLEDER was used as inoculum. Since the sludge was produced in a tannery, this inoculum was already adapted to chromium residues and collagen as substrate.

### 2.2 Pre‐treatment of the chromium shavings

In order to denature the materials and promote the waste degradation and biogas production, different heat and mechanical pre‐treatment techniques were tested. Extrusion, a classical technique from the polymer industry, and a continuous hydrothermal treatment, which is commonly used to plastify wood for the manufacture of wood composites, were used to pre‐treat and denature the chromium shavings. While extrusion affects the material by heat, mechanical shear, and pressure, the hydrothermal treatment is based on heat and steam pressure only. During the process, temperatures higher than the denaturation temperature were achieved in order to enable enzymatic degradation to produce biogas.

**PRACTICAL APPLICATION**

This work presents biogas production results for pre‐treated chromium leather shavings in a continuous reactor. This waste is available in great quantities in tanneries and is normally disposed of through landfill or incineration processes. However, chromium leather shavings are not utilized industrially to produce biogas because their degradation is slow and leads to low biogas yields. The use of pre‐treatments improves the digestion of the waste and could enable its use to produce biogas in an industrial environment in order to generate energy and reduce waste. Most large‐scale industrial digesters operate in the continuous mode; therefore, continuous tests trials are essential to develop this method industrially. However, hitherto there is no published work on continuous reactors for this material. The results of this study prove the feasibility of using pre‐treated chromium leather shavings to produce biogas in the industry.

#### 2.2.1 Extrusion

Extrusion was performed on chromium shavings with a co‐rotating twin screw‐extruder Werner & Pfleiderer ZSK 25 at 100°C in a continuous process. This extrusion process starts by feeding the sample from a hopper into the barrel of the extruder. The material is gradually degraded by the mechanical energy generated by turning screws and by heaters arranged along the barrel. The conversion of mechanical energy into heat makes it possible to use this process even under the denaturation temperature of chromium shavings (105 to 110°C). The extrusion of chromium shavings resulted in a powdered material with a water content of about 15%. The process takes approximately 3 min.

#### 2.2.2 Hydrothermal treatment

The chromium shavings were subjected to hydrothermal treatment through a continuous autoclave system attached to a refiner (Andritz CPH 12‐1) at the Institut für Holztechnologie (Dresden, Germany). Usually, this equipment is used to plastify wood chips but it is also adequate to process a variety of organic materials. The process was carried out at 140 and 150°C in saturated steam. The material was dosed into a digester in which it was denatured with steam under pressure. The pre‐treatment time was around 45 s.

The pre‐treated samples appeared like a dough with a high water content of about 80%. The direct use of these samples in the biogas trials was not possible due to their high water content. Therefore, drying and manual grinding of the material was necessary.

### 2.3 Characterization of the pre‐treated shavings

The pre‐treated materials were characterized regarding their inorganic matter 15, chromic oxide content 16, and collagen content by determination of the hydroxyproline content 17. Thermal profiles in fully hydrated state of the materials were taken using differential scanning calorimetry (DSC 1 STARe System Mettler Toledo) to verify that the pre‐treated materials were completely denatured. The enthalpy calculated from this method represents the necessary energy to break down the hydrogen bonds that stabilize the triple helix. If no enthalpy was observed, there is no triple‐helical collagen present in the sample. Results were compared with the values found for the untreated chromium shavings.

### 2.4 Biogas production trials

Continuous fermentation tests were performed according to the guideline VDI 4630 14. The continuous reactors consisted of a 20 L gastight stirred tank with infeed and outlet, and a gas offtake connection to collect the formed biogas. Temperature was kept under mesophilic conditions (37°C ± 2°C) using a circulation thermostat (Huber CC‐202C), and the biomass was constantly mixed at 50 rpm using a paddle stirrer (Heidolph RZR 2041). The biogas formation in norm litres per kg of sludge (wet basis) (L kg^−1^ d^−1^) was measured daily using a drum‐type gas meter (Ritter TG05/5), and the quality of the gas was measured using an electronic analyser (OPTIMA 7 BIOGAS) once every two days excluding weekends. Mesophilic anaerobic sludge from the SÜDLEDER tannery was used as inoculum.

At the beginning of the test, the reactor was filled with approximately 20 kg of inoculum (wet basis). Substrate was added once every two days excluding weekends via a dip tube located at the head end of the reactor, starting at a loading rate of substrate per kg of sludge of 0.5 g kg^−1^ d^−1^ (substrate mass in organic dry matter). After the daily methane production was constant, the loading rate was raised by 0.5 units and the process was repeated until the gas production no longer increased.

Once a week a sample of biomass was taken for characterization regarding its pH, inorganic matter 15, chromic oxide content 16, collagen content 17, chemical oxygen demand (COD) and ammonia content with Spectroquant® Cell Test Kits (Number 1.14541.0001 and 1.14739.0001 respectively). The biomass was also analysed chromatographically by high performance liquid chromatography (Shimadzu prominence Serie 20, equipped with a refractive index detector RID‐10A and a photodiode array detector SPD‐M20A) to determine its volatile fatty acids content.

The biogas formation measurements and the methane quantity were used to calculate the substrate degradation through a COD balance with Equation (1) 14. Using this method it is possible to determine the degradation level of the substrates through biogas and the substrate quantity data fed.

(1) $$\begin{array}{c} \mathrm{COD}\;\mathrm{degree}\;\mathrm{of}\;\mathrm{degradation}\;=\;\frac{V_{\mathrm{Gas}}\cdot \;x_{\mathrm{CH}4}}{320\;\cdot \;m_{\mathrm{substrate}}\cdot \;\mathrm{CO}D_{\mathrm{substrate}}}\% \end{array}$$

Where *V*_Gas_ is the volume of biogas in mL d^−1^ (norm), x_CH4_ is the amount of methane, *m*_substrate_ is the substrate addition in g d^−1^ (organic dry matter), and COD_substrate_ is the theoretical substrate's COD (1.124 g_O2_ g^−1^ in organic dry matter). The balance was made using the cumulative volume of methane produced in the reactors and the cumulative mass of added substrate starting on the first day of digestion.

## 3 RESULTS AND DISCUSSION

### 3.1 Characterization of the pre‐treated shavings

The characterization of the pre‐treated samples gives important information for the biogas trials. Only organics are capable of producing biogas and, therefore, it is important to quantify the organic and inorganic content. The characterization of the samples is presented in Table 1.

**Table 1:** Characterization of the untreated and pre‐treated chromium shavings

	Organic matter (%)^a^	Chromium (%)^a^^,^ b	Collagen (%)^a^	Denaturation enthalpy (J g^−1^)^c^
Chromium shavings	88.8 ± 0.1	4.6 ± 0	77 ± 0.6	61.8 ± 0.6
Extruded shavings	88.9 ± 0	4.6 ± 0	74.2 ± 0.9	0
Shavings treat. hydrothermally	89.5 ± 0.4	4.4 ± 0	74.2 ± 0.8	0

a Dry basis; mean ± SD, *n* = 3.

b Measured as chromium oxide.

c Mean ± SD, *n* = 2.

The organic matter in the samples remains the same after pre‐treatment. This is important because the organics must be preserved for producing biogas in the anaerobic digestion. The collagen content is also barely unchanged after pre‐treatment. This protein is the main component of the chromium shavings, only about 12% of the shavings are different types of organics, for instance fats. Therefore, collagen is the most important parameter to calculate the substrate degradation after anaerobic digestion. DSC results show that the collagen in the chromium shavings was completely denatured by pre‐treatment. Consequently, chromium shavings are more easily accessible to enzymatic degradation. This is explored in more detail in a previous work 7.

Almost half of the inorganic part of the samples is chromium oxide. Chromium also remains the same after pre‐treatment. Other inorganics in the samples come from the chemicals used in tanneries.

### 3.2 Biogas production trials

#### 3.2.1 Productivity of the reactors

**Figure 1:**
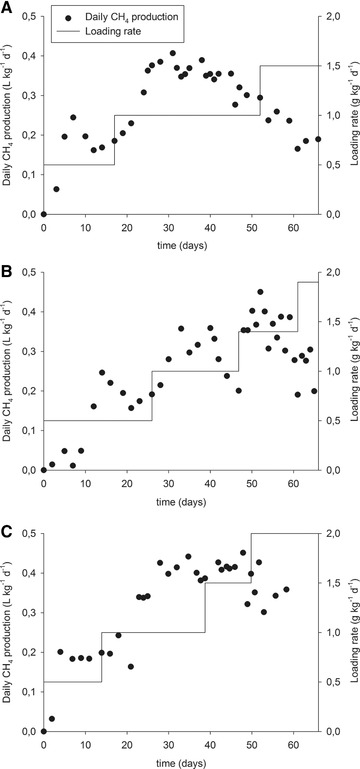
Time plot of continuous fermentation of the untreated chromium shavings (A), extruded shavings (B), and shavings treated hydrothermally (C)

The pre‐treated samples were tested as substrates for biogas production in a continuous reactor and the results were compared with the performance of the untreated chromium shavings. Figure 1 shows the time plot of digestion for the studied substrates.

Two of the biogas trials showed a drop in the daily methane production along digestion, indicating technical problems. The methane production drops can be seen on the 7th day of digestion for the reactor fed with extruded shavings (Figure 1B) and on the 21st for the reactor fed with shavings treated hydrothermally (Figure 1C). The former was caused by an oxygen infiltration on day 5 due to the rupture of the dip tube used to feed the reactor; the latter by an agitation failure, which resulted in the uneven heating of the reactor. Even though there is a drop in the methane production, the system recovered its former stability and appeared to function normally after a few days. The reactor fed with shavings treated hydrothermally needed only two days to recover, and the reactor fed with extruded shavings needed five days, that is more time to recover probably because the ingress of oxygen occurred very early in the digestion process. The daily methane production became very unstable for the extruded shavings when using loading rates higher than 1.4 g kg^−1^ d^−1^ and for the shavings treated hydrothermally for loading rates of 2 g kg^−1^ d^−1^.

The production of a second batch of extruded shavings was necessary to feed the reactor starting on the 42nd day. This second batch of the substrate was not completely denatured because the use of the same extruder was not possible. DSC results still showed a denaturation enthalpy of 12.6 J g^−1^ compared to 61.8 J g^−1^ for untreated chromium shavings (Table 1).

Pre‐treatment allowed the use of a higher loading rate and increased the maximum daily methane production. The trial with untreated chromium shavings (Figure 1A) had to be stopped at a loading rate of 1.5 g kg^−1^ d^−1^ but the extruded shavings (Figure 1B) could be tested up to a loading rate of 1.9 g kg^−1^ d^−1^ and the shavings treated hydrothermally (Figure 1C) up to 2.0 g kg^−1^ d^−1^. The untreated chromium shavings reached the maximum daily methane production, 0.41 L kg^−1^ d^−1^, on day 31 of digestion with a loading rate of 1.0 g kg^−1^ d^−1^. The extruded shavings reached a higher value, 0.45 L kg^−1^ d^−1^, on day 52 of digestion but with a loading rate of 1.4 g kg^−1^ d^−1^. Similarly, for the shavings treated hydrothermally, the maximum daily methane production was 0.45 L kg^−1^ d^−1^, on day 48 of digestion with a loading rate of 1.5 g kg^−1^ d^−1^.

The production of methane was low at the beginning of the digestion. The composition of the produced biogas presented less than 40% of methane until the third day of digestion for the reactor fed with untreated chromium shavings, until the fifth day for the extruded shavings reactor, and until the second day for the reactor using shavings treated hydrothermally. After that, methanation increased reaching its highest level of about 68% for the three tested substrates at the first tested loading rate. The reactor fed with extruded shavings showed the longer delay in methanation among the studied substrates. This was probably caused by the ingress of oxygen at the beginning of the anaerobic digestion trial.

Trials for all studied substrates presented a reduction of biogas quality at the loading rate in which they had their maximum daily methane production. The trial with untreated chromium shavings and the trial with shavings treated hydrothermally presented on average 62% of methane at this this loading rate and the trial with extruded shavings presented on average 55% of methane. Despite this, the quality values were stable. It was possible to produce more biogas but the methanogenesis started to be inhibited when increasing the load of substrate. At the end of the anaerobic digestion, the quality dropped indicating failure of the digestion.

No studies covering the anaerobic digestion of chromium leather waste in continuous trials could be found for comparison. Some authors studied the digestion of tannery waste in continuous 18, 19 or semi‐continuous mode 20, 21, 22 but the material was always collected from tanneries prior to the tanning step and this is known to be already in use industrially 3. These studies mainly used fleshings as substrate, which are also not suitable for comparison due to a high fat content.

#### 3.2.2 Inhibition of digestion

**Figure 2:**
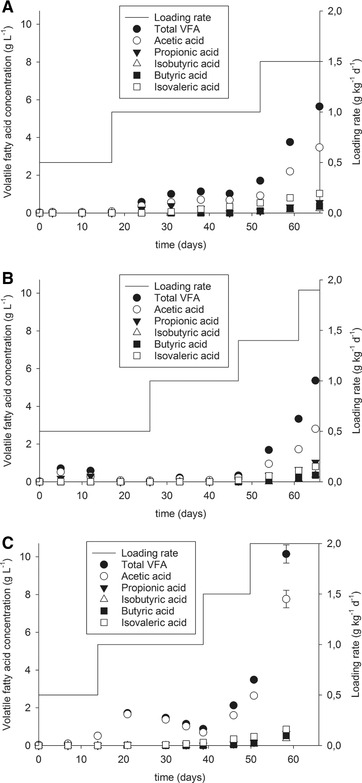
Volatile fatty acid concentrations along the anaerobic digestion of untreated chromium shavings (A), extruded shavings (B), and shavings treated hydrothermally (C)

In order to monitor the reactor stability and possible inhibitions, biomass samples were collected weekly and analyzed regarding their concentration of volatile fatty acids. Figure 2 shows the results for the three anaerobic reactors.

Volatile fatty acids are intermediate products resulting from anaerobic digestion. However, they could inhibit the methane production at high concentrations 12. To avoid failure of a fermenter, the total concentration of volatile fatty acids should be lower than 4 g L^−1^. The acetic acid concentration should be lower than 3 g L^−1^, the isobutyric acid concentration lower than 0.5 g L^‐1^, and the propionic acid concentration lower than 1 g L^−1^ 23 but a concentration of propionic acid higher than 0.3 g L^−1^ is enough to disturb the anaerobic digestion 24.

The reactor fed with untreated chromium shavings (Figure 2A) showed a stable total volatile fatty acid concentration for a loading rate up to 1 g kg^−1^ d^−1^ but the concentration increased at a loading rate of 1.5 g kg^−1^ d^−1^ to more than 4 g L^−1^, corresponding to the drop in daily methane production. For the final collected sample, the acetic acid concentration was 3.5 g L^−1^ and the propionic acid concentration reached 0.6 g L^−1^ indicating a complete failure of the reactor at this loading rate.

Similarly, for the extruded shavings (Figure 2B) as substrate, the total volatile fatty acid concentration was low for a loading rate up to 1 g kg^−1^ d^−1^. There was a small increase of acid concentration at the first loading rate, corresponding to the ingress of oxygen registered for this reactor but the system recovers its former stability and the acid concentration drops again. The acid concentration started increasing at a loading rate of 1.4 g kg^−1^ d^−1^ without reaching an inhibitory concentration, which could explain the unstable daily methane production values at this loading rate. Finally, at the last loading rate, the total acid concentration reached 5.4 g L^−1^ and the propionic acid concentration reached a value of 1 g L^−1^ indicating the failure of the reactor. The acetic acid and isobutyric acid concentrations were also high in this last sample but without reaching an inhibitory concentration.

The reactor fed with shavings treated hydrothermally (Figure 2C) also showed a small increase of the volatile fatty acid concentration due to the agitation failure at a loading rate of 1 g kg^−1^ d^−1^. Again, the reactor recovers stability and the acid concentration drops. Otherwise, the reactor showed stable volatile fatty acid concentrations. The concentration only increased in the last collected biomass sample at a loading rate of 1.5 g kg^−1^ d^−1^. Inhibition began at the last loading rate and daily methane production dropped. The total volatile fatty acid concentration increased to up to 10.1 g L^−1^, the acetic acid concentration to up to 7.8 g L^−1^, propionic acid to up to 0.6 g L^‐1^, and isobutyric acid to up to 0.4 g L^−1^.

The chromium content could also cause inhibition of the methane production in the reactors. Cr^3+^ ions could inhibit the methanogenic archaea without affecting the acidogenic bacteria, resulting in the accumulation of volatile fatty acids. Chromium could also have other negative effects on the anaerobic digestion such as a decrease in the total gas production rate, a fall in pH, a decrease in the percentage of methane in the gas produced, and an accumulation of organic matter 25. The pH and chromium content of the collected biomass samples was analysed and results are shown in Table 2.

**Table 2:** pH and chromium content of the samples collected along the continuous digestion

Chromium shavings			
Time (days)	Loading rate (g kg^−1^ d^−1^)	pH	Chromium (%)^a^
0	0.5	8.1	1.1
3			1.0 ± 0.0
10		7.9	1.4 ± 0.1
17		7.9	1.6 ± 0.1
24	1.0	7.9	1.9 ± 0.0
31		8.0	2.4 ± 0.1
38		8.1	2.7 ± 0.0
45		8.1	3.5 ± 0.0
52		8.1	3.3 ± 0.1
59	1.5	8.1	3.9 ± 0.1
66		8.0	4.3 ± 0.1

Extruded shavings			
0	0.5	8.3	1.0 ± 0.0
5		7.8	1.3 ± 0.1
12		7.9	1.6 ± 0.0
19		8.0	2.0 ± 0.1
26		7.9	2.5 ± 0.0
33	1.0	8.0	3.1 ± 0.1
40		8.1	3.5 ± 0.1
47		8.0	3.8 ± 0.1
54	1.4	8.0	4.7 ± 0.1
61		8.1	4.9 ± 0.4
65	1.9	8.0	4.5 ± 0.0

Shavings treated hydrothermally			
0	0.5	8.1	1.2 ± 0.0
7		8.0	1.6 ± 0.0
14		8.0	1.7 ± 0.0
21	1.0	7.8	2.3 ± 0.0
30		8.1	2.8 ± 0.1
35		8.1	3.1 ± 0.0
39		8.1	3.1 ± 0.0
46	1.5	8.1	3.5 ± 0.1
51		8.1	4.7 ± 0.0
58	2.0	8.0	4.7 ± 0.0

a Dry basis; mean ± SD, *n* = 2; measured as chromium oxide.

The pH of the biomass during digestion was very stable, between 7.8 and 8.1, causing no disturbance in the system. A pH between 6.5 and 8 is needed for acetogenic bacteria and methanogenic archaea in the anaerobic digestion, therefore, the pH of the reactor must be in this range 26.

At the end of the trials, a total chromium content of almost 5% (measured as chromium oxide) was achieved in all reactors, which appears not to affect digestion. The inoculum for the reactor fed with untreated chromium shavings had a chromium content of 324 mg L^−1^, the extruded shavings reactor had a chromium content of 233 mg L^−1^, and the reactor fed with shavings treated hydrothermally had a content of 369 g L^−1^. The bacteria present in the initial sludge were already adapted to high quantities of chromium prior to digestion. The determination of a chromium content limit would only be possible with a long‐term trial at an appropriate loading rate, without other inhibitions.

**Figure 3:**
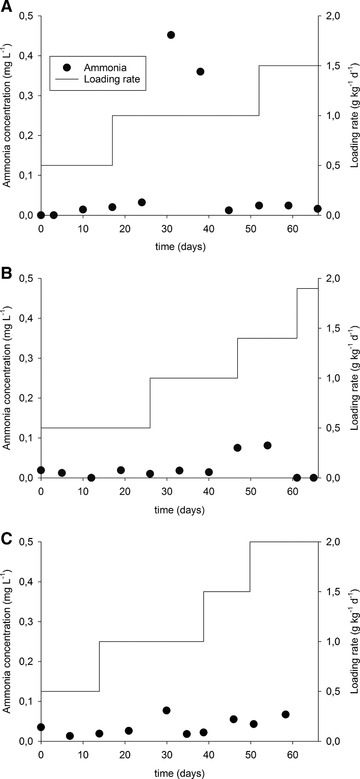
Concentration of ammonia found in the collected biomass at different reaction times for the reactor fed with untreated chromium shavings (A), extruded shavings (B), and shavings treated hydrothermally (C)

Bovine hide collagen, the main component of the studied substrates, is a nitrogen‐rich substrate with a C/N ratio of 3.1 and there is the possibility of inhibition by ammonia. The ammonia content in the biomass of the reactors was monitored along the anaerobic digestion. Figure 3 presents the concentration of ammonia results. The sample point 0 represents the ammonia content of the inoculum before the digestion.

The inhibition level of ammonia is known to be 1.5 to 3.0 g NH_4_‐N L^−1^ 27. Ammonia is present in the biomass in a concentration below 0.5 g NH_4_‐N L^−1^ in the three continuous trials and caused no problem to the anaerobic digestion. The ammonia generated by fermentation of the amino acids from collagen did not accumulate presumably because it was used for bacterial growth.

#### 3.2.3 Degradation of the substrate

The degradation of the substrate was evaluated analysing the biomass samples collected weekly along the anaerobic digestion. Table 3 shows the characterization of the collected biomass samples. The samples were analysed regarding their collagen, organic matter, and chromium content.

**Table 3:** Biomass characterization of the samples collected along the continuous digestion

Chromium shavings			
Time (days)	Loading rate (g kg^−1^ d^−1^)	Collagen (%)^a^	Organic matter (%)^a^
0	0.5	2.9 ± 0.3	39.5 ± 1.4
3		4.3 ± 0.9	41.4 ± 1.2
10		4.4 ± 0.4	41.2 ± 1.8
17		5.8 ± 0.6	38.7 ± 0.2
24	1.0	6.3 ± 0.7	41.1 ± 0.3
31		5.1 ± 0.4	40.8 ± 0.0
38		3.6 ± 0.4	40.2 ± 0.3
45		2.5 ± 0.1	40.3 ± 0.2
52		2.3 ± 0.1	41.3 ± 0.2
59	1.5	2.6 ± 0.2	44.6 ± 0.3
66		3.3 ± 0.0	45.4 ± 0.1

Extruded shavings			
Time (days)	Loading rate (g kg^−1^ d^−1^)	Collagen (%)^a^	Organic matter (%)^a^
0	0.5	2.7 ± 0.3	38.3 ± 0.3
5		3.2 ± 0.1	38.4 ± 1.2
12		3.3 ± 0.1	38.5 ± 0.3
19		3.6 ± 0.1	39.6 ± 1.4
26		3.4 ± 0.1	37.9 ± 0.1
33	1.0	3.4 ± 0.3	40.2 ± 0.5
40		4.5 ± 0.2	40.3 ± 0.2
47		6.5 ± 0.3	42.0 ± 0.1
54	1.4	3.4 ± 0.1	43.7 ± 0.2
61		3.1 ± 0.1	44.4 ± 0.1
65	1.9	2.8 ± 0.0	46.9 ± 0.1

Shavings treated hydrothermally			
Time (days)	Loading rate (g kg^−1^ d^−1^)	Collagen (%)^a^	Organic matter (%)^a^
0	0.5	3.3 ± 0.0	37.4 ± 0.2
7		3.7 ± 0.0	39.1 ± 0.3
14		4.0 ± 0.1	37.9 ± 0.1
21	1.0	5.6 ± 0.0	40.7 ± 0.3
30		4.8 ± 0.3	39.8 ± 0.6
35		5.1 ± 0.1	39.8 ± 0.4
39		5.2 ± 0.1	40.6 ± 0.5
46	1.5	4.9 ± 0.1	42.3 ± 0.3
51		5.0 ± 0.0	45.3 ± 0.4
58	2.0	4.0 ± 0.1	48.0 ± 0.1

a Dry basis; mean ± SD, *n* = 3.

An increase in the organic matter content in the collected sludge would indicate the accumulation of unprocessed substrate and, therefore, inefficiency of the process. As seen in Table 3, the reactor fed with untreated chromium shavings showed a constant value of organic matter content (around 40%) for most of the time. An increase to 45% was detected at the end of the trial at a loading rate of 1.5 g kg^−1^ d^−1^, indicating that the reactor was overloaded. The methane production is very low and the added substrate accumulates.

The reactor fed with the extruded shavings had an organic matter content of around 39% at a loading rate of 0.5 g kg^−1^ d^−1^. At a loading rate of 1.0 g kg^−1^ d^−1^ the organic matter content remained constant at 40% and showed a slight increase to 42% at the end of this rate. In a next step, an increase in organic matter to around 44% was observed at a loading rate of 1.4 g kg^−1^ d^−1^. Finally, in the last step, the organic matter content increased to 47% at a loading rate of 1.9 g kg^−1^ d^−1^. With regard to the shavings treated hydrothermally, the reactor started with around 38% of organic matter at the first loading rate. Progression to the next step increased the organic matter content of the reactor to about 40%. The loading rate of 1.5 g kg^−1^ d^−1^ resulted in an increase to 45% and the last loading rate led to 48%. The increase of organic matter at the last loading rate when methane production slows indicates accumulation.

At the end of digestion, the collagen content was almost as low as it was prior to adding substrate at the beginning of the process for all reactors, showing that collagen was degraded and that there is no accumulation of collagen in the reactor. Therefore, the collagen of the added substrate was metabolized. However, the inorganic part of the samples accumulated, and the chromium content in the reactor increased.

Although a second batch of extruded shavings was used, which were not completely denatured, the reactor accomplished degradation of the substrate. When the reactor was shut down, it contained 33.7 g of collagen (dry basis), which represents a final degradation of 96.5% of all of the added collagen (953.8 g of added collagen). The reactor fed with untreated chromium shavings contained 45.6 g of collagen (dry basis) at shutdown, equating to 95.5% of degradation (1014.4 g of added collagen) and the reactor fed with shavings treated hydrothermally showed a final amount of collagen of 58.3 g (dry basis) meaning that 95.3% of the added collagen were degraded (1233.4 g of added collagen).

**Figure 4:**
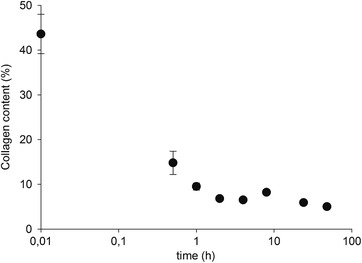
Collagen content of the biomass samples collected after substrate feeding (logarithmic scale of the time)

The degradation of collagen after one feed was further studied for the reactor fed with shavings treated hydrothermally. After feeding the reactor with substrate on day 51 of the digestion, at loading rate of 2.0 g kg^−1^ d^−1^, small biomass samples were collected from the reactor at different times along two days for collagen content analyses in triplicate. Figure 4 shows the collagen content results for the collected biomass samples.

Before feeding, the reactor presented a collagen content of 5%. Shortly after feeding, the collagen content rose to about 44%, a very high value probably due to the inhomogeneity of the substrate, which can be seen in the high variance of the results. The substrate needed more time to be solubilized in the biomass. After only 2 h, the collagen content in the biomass was almost as low as before substrate feeding, indicating that most of the collagen had been rapidly degraded. The collagen content dropped to 5%, the same collagen content detected before feeding, after 48 h. That leads to the conclusion that there is efficient degradation of collagen notwithstanding the observed accumulation of organic matter (Table 3).

The organic matter present in the final biomass is formed from organics from the inoculum and intermediate products from the hydrolysed substrate, such as volatile fatty acids, amino acids, and peptides. In a previous work 7, it was shown that the anaerobic digestion of inoculum without adding substrate leads to a final biomass of about 40% of organic content from which the conclusion can be drawn that part of the initial organic matter will remain unprocessed. The increase in organic matter content along the digestion is a consequence of the accumulation of unprocessed substrate in the form of intermediate products, which are not transformed into biogas probably due to inhibition of the anaerobic bacteria.

**Figure 5:**
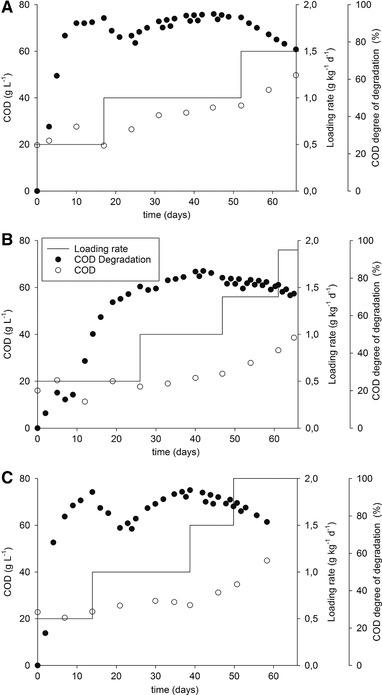
Cumulative COD degree of degradation during digestion and COD in the biomass samples collected along the anaerobic digestion of untreated chromium shavings (A), extruded shavings (B), and shavings treated hydrothermally (C)

The substrate degradation was evaluated through a COD balance calculated with Equation (1). The cumulative COD degree of degradation shows degradation of substrate in the process as a whole and indicates that almost all of the substrate was successfully degraded. Furthermore, the biomass samples collected were analyzed regarding COD as another indicative of organics in the biomass. The results are shown in Figure 5.

The maximum COD degree of degradation for the trial using untreated chromium shavings as substrate (Figure 5A) was 94.9% at the loading rate that showed the highest daily methane production for this substrate (1.0 g kg^−1^ d^−1^). First, there is a reduction of COD degree of degradation and later an increase, indicating that the system needs to adapt to higher loads before starting production at its maximum capacity. For the same loading rate, COD increases slowly. In the last loading rate, COD rises steeply and the COD degree of degradation declines.

The reactor fed with shavings treated hydrothermally (Figure 5C) showed a maximum cumulative COD degree of degradation of 92.5% at a loading rate of 1.5 g kg^−1^ d^−1^, the loading rate for the maximum daily methane production reached in this trial. There is a drop of COD degree of degradation at a loading rate of 1.0 g kg^−1^ d^−1^ because the system needed to adapt to a new loading rate after the rise from 0.5 to 1.0 g kg^−1^ d^−1^, which caused a slight drop in degradation, and the agitation failure intensified the drop. The COD values in the biomass samples were stable for a loading rate of up to 1.0 g kg^−1^ d^−1^, increased slightly for the next step, and steeply at the last step.

The reactor fed with extruded shavings takes longer than the other trials to reach high COD degrees of degradation (Figure 5B) due to the ingress of oxygen. The highest COD degree of degradation of 83.8% was achieved at a loading rate of 1.0 g kg^−1^ d^−1^ and not for the next step as expected, when the maximum daily methane production was reached. The value was lower than that for the other reactors, probably due to the instability caused by the increasing volatile fatty acids recorded for a loading rate of 1.4 g kg^−1^ d^−1^. COD values started increasing at a loading rate of 1.0 g kg^−1^ d^−1^ and they became steeper for every subsequent loading rate step.

Considering that the highest daily methane production for the untreated chromium shavings was reached at a loading rate of 1.0 g kg^−1^ d^−1^, the conclusion can be drawn that this is the most appropriate loading rate for this material. The reactor fed with extruded shavings showed the highest daily methane production at a loading rate of 1.4 g kg^−1^ d^−1^ proving to be the most suitable loading rate for the extruded sample among the studied rates. Finally, the reactor fed with shavings treated hydrothermally reached its highest daily methane production at a loading rate of 1.5 g kg^−1^ d^−1^ showing that this rate should be aimed at. Accumulation of organic matter and the increasing concentrations of volatile fatty acids verified in the collected biomass samples for the last tested loading rates lead to the same conclusion. Nevertheless, long‐term trials of one year or more should prove this observation.

Pre‐treatment of the chromium shavings allows for a higher loading rate of substrate in the reactor and a higher daily methane production. This increases the capability of reducing solid waste and the generation of energy. Consequently, the feasibility of chromium shavings to be digested for producing biogas in tanneries increases. It is also possible to digest untreated chromium shavings in continuous systems to produce methane but using a low loading rate. However, an explanation for this observation is still missing and this conflict with the common doctrine that untreated chromium shaving cannot be digested in biogas reactors. An economical evaluation to verify if the pre‐treatment costs are compensated by the energy gains should be done. However, reduction of the disposal of final waste, which would otherwise generate costs, makes this method very attractive for industry purposes.

## 4 CONCLUDING REMARKS

The production of biogas in continuous reactors was tested for two different pre‐treated chromium shavings and untreated chromium shavings. Pre‐treatment was carried out to denature the stable collagen structure of chromium shavings in order to enhance anaerobic digestion when using this waste as substrate to produce biogas.

The reactors fed with pre‐treated shavings showed a higher methane production than those using untreated chromium shavings as substrate. For pre‐treated shavings a loading rate could be used which was 40 to 50% higher than that for untreated chromium shavings, and the maximum daily methane production could be increased by almost 10%. A higher loading rate leads to a more economical process, and it can be expected that, on an industrial scale, the loading rate could be increased by 40 to 50% through pre‐treatment of the chromium shavings or, in contrast, the reactor volume for a given loading rate could be decreased.

The biogas production in continuous reactors using chromium shavings as substrate was improved by the use of pre‐treatments. This increases the feasibility of digesting chromium shavings to produce biogas and energy in tanneries.

## CONFLICTS OF INTEREST

The authors have declared no conflict of interest.
